# Multifocal brain abscesses caused by invasive *Streptococcus intermedia*: A case report

**DOI:** 10.3389/fneur.2022.893627

**Published:** 2022-08-12

**Authors:** Lin Yao, Sansong Chen, Zuan Yu, Tao Yu

**Affiliations:** Department of Neurosurgery, The Translational Research Institute for Neurological Disorders, The First Affiliated Hospital of Wannan Medical College (Yijishan Hospital), Wuhu, China

**Keywords:** multiple brain abscesses, *Streptococcus intermedia*, meta-genomic next-generation sequencing, case report, cerebrospinal fluid

## Abstract

Multifocal brain abscesses caused by invasive *Streptococcus intermedia* are relatively rare. Here, we present a 67-year-old male was admitted to the hospital for unconsciousness and fever. The computed tomography (CT) examination showed multiple intracranial space-occupying and “cavity-like” changes in the right lower lung. The examination of cerebrospinal fluid (CSF) was consistent with typical bacterial meningitis, CSF analyses revealed leukocytosis (10,300 × 10^6^/L), elevated protein levels (140.39 mg/dL), decreased glucose levels (0.27 mmol/L), and normal chloride concentration level (120.2 mmol/L), however, pathogens were not detected in the cultures. Then, the CSF and sputum samples were analyzed using meta-genomic next-generation sequencing (mNGS), and *S. intermedia* was detected in both samples. We adjusted the use of antibiotics according to the results of mNGS in time. After anti-infective treatment, the patient achieved good treatment results in a very short time. This case highlights the mNGS can identify pathogens of brain abscess, and provide strong evidence for clinical diagnosis and treatment strategy.

## Introduction

Brain abscess is often a localized brain infection caused by the spread of nearby infections, such as otitis, mastoiditis, sinusitis, neurosurgery or traumatic brain injury, and blood spread (1). It has a prevalence of ~0.9 per 10,000 people per year, with a 1-year mortality rate of ~20%. Common clinical symptoms of brain abscess include headache, fever, vomiting, cramps, and focal neurological deficits (2).

*S. intermedia* exists mainly exists in the human oral cavity, throat, and gastrointestinal tract. *S. intermedius* can cause gingival abscess and aspiration pneumonia, leading to sepsis, endocarditis, and lung abscesses. *S. intermedia* has been reported to cause brain abscesses and often coexist with anaerobic bacteria (3, 4). Herein, we present a case of multifocal brain abscess that showed the diagnosis and treatment of the patient.

## Case presentation

**Figure 1:**
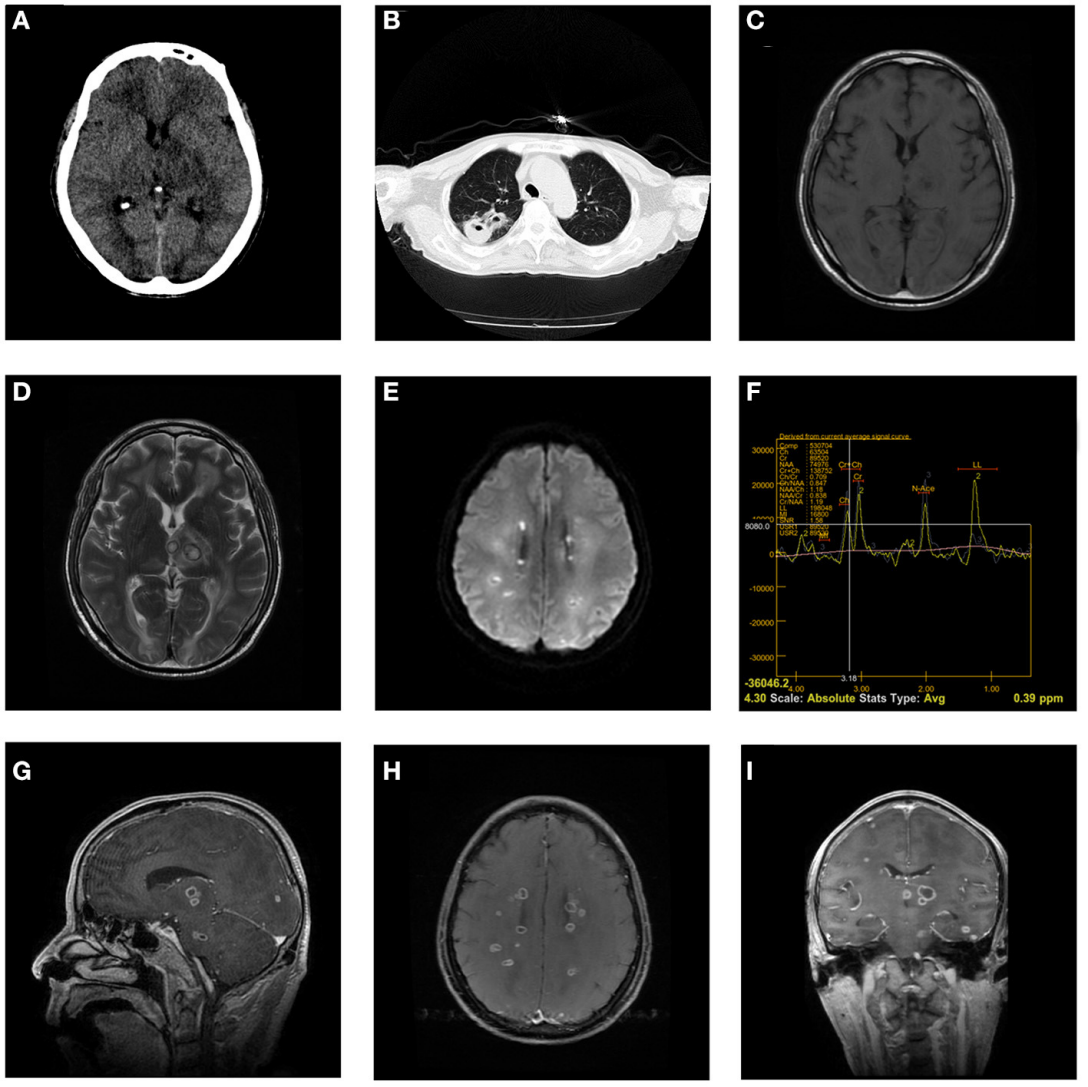
Head CT on admission shows multiple annular nodules in the left thalamus, and the edema is obvious **(A)**. The chest CT shows a space-occupying lesion in the cavity in the upper lobe of the right lung **(B)**. The head MRI on May 12 showed that scattered multiple signals can be seen in the bilateral cerebral hemispheres, thalamus, and brainstem, which were low signal on the T1 weighted image and high signal on the T2 weighted image and diffusion-weighted imaging **(C–E)**. Magnetic Resonance Spectroscopy (left thalamus) showed that the Cho peak, Cr peak, and NAA peak decreased, the Cho/Cr ratio was 0.709, the Cho/NAA ratio was 0.847, and the LL peak increased significantly **(F)**. Brain abscess was significantly enhanced **(G–I)**.

A 67-year-old male was admitted to our hospital due to unconsciousness and fever. The patient had fever symptoms 3 days before admission, the highest temperature was 39.6°C, and no special treatment was given. The patient had a history of drunkenness and aspiration 1 week before admission. One day before admission, the patient suddenly lost consciousness with quadriplegia. Physical examination: the patient was in a deep coma; the diameter of the bilateral pupils was 2 mm; the Glasgow score was E1V1M3; and the muscle strength of the limbs was grade 2. Blood analyses revealed evidence of leukocytosis (33.6 × 10^9^/L) and elevated C-reactive protein (CRP) levels (199.80 mg/L), neutrophils percentage (93.80%), and procalcitonin range (1.58 ng/ml). Head computed tomography (CT) showed multiple intracranial space-occupying edema, and chest CT showed a right upper lobe space occupying the cavity. Brain magnetic resonance imaging (MRI) showed multiple divergent intracranial abscesses, which were low signal on the T1 weighted image and high signal on the T2 weighted image and were significantly enhanced. On the day of admission, the patient had difficulty breathing and was given mechanical ventilation with tracheal intubation (Figure 1). On day 3, a tracheotomy was performed. The patient had no history of tuberculosis, sputum tuberculosis and tuberculosis infection T cell detection was negative.

To determine the patient's intracranial infection, we performed a lumbar puncture on the third day after admission. Cerebrospinal fluid (CSF) analyses revealed leukocytosis (10,300 × 10^6^/L), elevated protein levels (140.39 mg/dL), decreased glucose levels (0.27 mmol/L), and normal chloride concentration level (120.2 mmol/L). However, pathogens were not detected in the cultures, and Gram staining assays were prepared from CSF, sputum, and blood samples. We used vancomycin (2 g every 12 h, ivgtt) combined with meropenem (1 g every 8 h, ivgtt) and ornidazole (0.5 g every 12 h, ivgtt) as an empirical anti-infection treatment. We used mNGS to identify pathogens in CSF and alveolar lavage fluid on 5th day after admission (Table 1). We stopped vancomycin and meropenem, then switched to ceftriaxone (2 g every 24 h, ivgtt) combined with amikacin (0.4 g every 12 h, ivgtt) for anti-infection treatment.

**Table 1:** List of macro genomic pathogens detected in alveolar lavage fluid and cerebrospinal fluid (CSF) samples on 5 days after admission.

**Sample**	**Gram staining**	**Genera**	**Sequence number**	**Species**	**Sequence number**	**Relative abundance (%)**	**Coverage (%)**
Alveolar lavage fluid	G^−^	Burkholderia	15,266	*Burkholderia cenocepacia*	1,295	3.72	16.42
	G^+^	Streptococcus	63,514	*Streptococcus intermedius*	10	0.0288	1.64
	G^−^	Pseudomonas	37	*Pseudomonas aeruginosa*	8	0.0230	0.0689
CSF	G^+^	Streptococcus	49	*Streptococcus intermedius*	24	39.34	0.19

The patient's consciousness and limb muscle strength gradually improved during the subsequent treatment. Two weeks after admission, the patient's body temperature was normal, and no more than 39°C. Celsius Routine blood leukocyte count, neutrophil ratio, and procalcitonin levels decreased gradually to normal levels. We performed lumbar puncture during hospitalization at the third week after admission, which showed that the patient's intracranial pressure was 180 mmH_2_O and the color of the CSF was light blood and clear; CSF analyses showed normal leukocyte count (15 × 10^6^/L), elevated protein levels (72.3 mg/dL), and normal glucose levels (4.37 mmol/L) and chloride concentration level (119 mmol/L). We then sequenced the CSF again at 4 weeks after admission, suggesting that *S. intermedia* could still be detected, but its relative abundance decreased from 39.34 to 5.62% (Table 2). We continued to use ceftriaxone in the treatment of intracranial infection until the patient's body temperature was normal for 2 weeks, we stopped all antibiotics at 6 weeks after admission (Table 3).

**Table 2:** List of macro genomic pathogens detected in cerebrospinal fluid (CSF) samples at 4 weeks after admission.

**Sample**	**Gram staining**	**Genera**	**Sequence number**	**Species**	**Sequence number**	**Relative abundance (%)**	**Coverage (%)**
**CSF**	G^+^	Streptococcus	11	*Streptococcus intermedius*	5	5.62	0.0403

**Table 3:** Timeline.

3 days before admission	Presented with fever symptoms, no treatment was given.
1 days before admission	Lost consciousness with quadriplegia
The day of admission	Tracheal intubation
	Improve laboratory examinations
	Vancomycin combined with meropenem and ornidazole as an empirical anti-infection treatment
3 days after admission	Lumbar puncture: CSF leukocyte count was 10,300 × 10^6^/L
	Tracheotomy was performed
4 days after admission	T-SPOT was negative
5 days after admission	The mNGS was used to in CSF and alveolar lavage fluid
7 days after admission	Changed antibiotics
2 weeks after admission	Fever symptoms improved
3 weeks after admission	Lumbar puncture: CSF leukocyte count was 15 × 10^6^/L
4 weeks after admission	The mNGS was used to in CSF again, relative abundance decreased
6 weeks after admission	Stopped antibiotics, and moved out of ICU
8 weeks after admission	The patient's consciousness became clear
10 weeks after admission	Discharged

**Figure 2:**
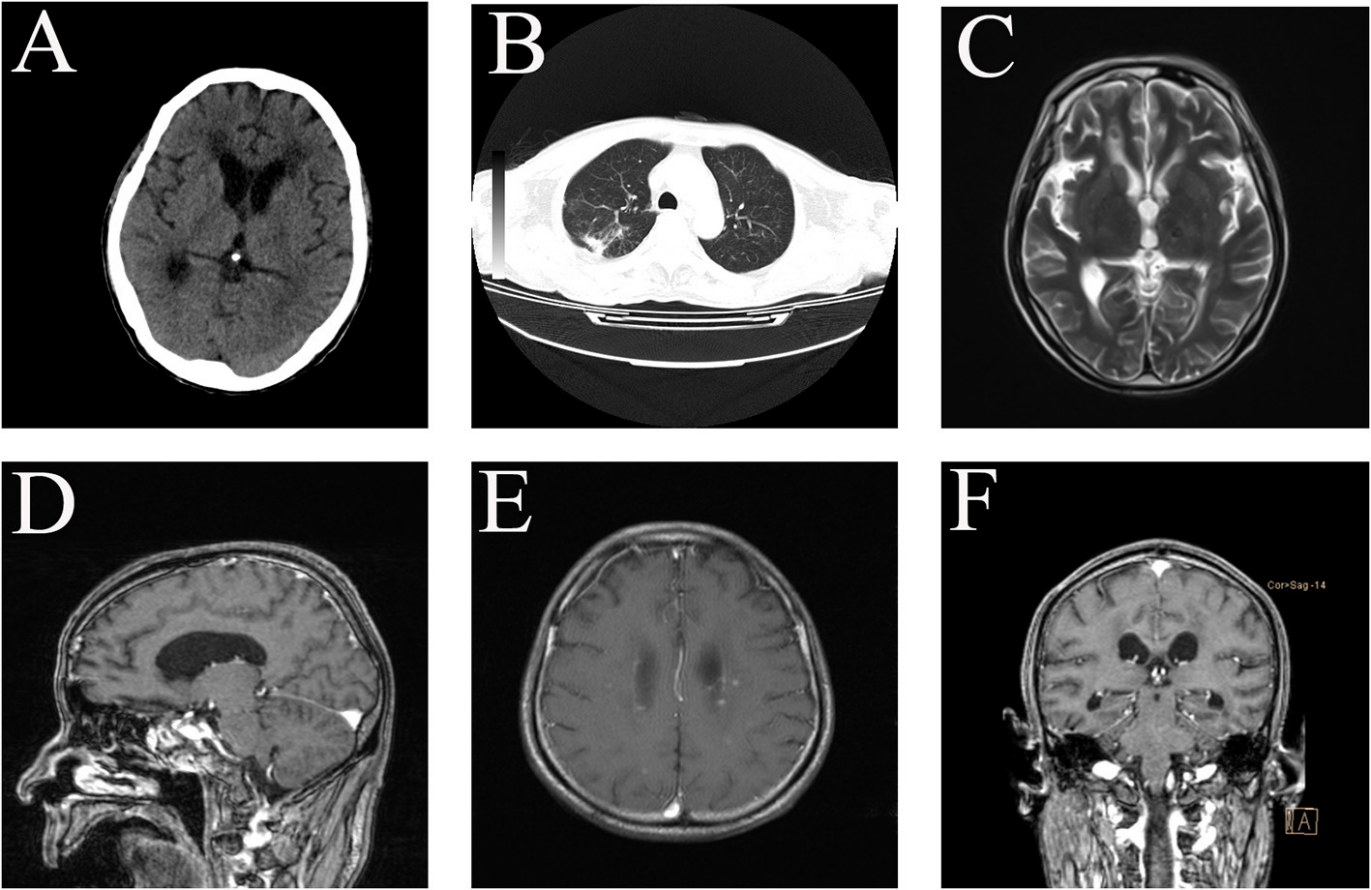
CT after 2 months of treatment. The size of the lesion and the degree of edema improved significantly **(A)**. The patient's upper right pneumonia is better than before **(B)**. The patient MRI in August 2021 showed that the abscess was significantly absorbed and improved **(C–F)**.

After 8 weeks, the patient's consciousness became clear, and the muscle strength of the limbs recovered to grade 4. CT showed that the size of the lesion and the degree of edema improved significantly, the patient's upper right pneumonia was better than before, and the patient was discharged at the 10 weeks after admission (Table 3). The 3-month follow-up head MRI showed that the brain abscess had basically disappeared (Figure 2).

## Discussion

Multiple intracranial lesions accompanied by cavitation changes in the lungs need to rule out lung cancer brain metastases and tuberculous brain abscesses. The main symptom of the patient was high fever. Combined with the patient's laboratory examination, the first consideration was inflammatory disease. Tuberculous brain abscesses and bacterial brain abscesses have similar clinical and conventional MRI findings, both manifest as ring-enhancing cystic space-occupying lesions, which require further differential diagnosis with the help of clinical manifestations, biopsy, pathology, etc (5). Combined with the patient's medical history, laboratory examination, and imaging examination, we considered that the patient had a bacterial brain abscess.

Regarding the choice of the treatment plan for brain abscess, it is necessary to comprehensively evaluate the patient's clinical status and abscess. In this case, most of the intracranial lesions were divergent, and most of the abscesses were <1 cm in diameter, most of which were in the functional area of the brain, and a stereotactic puncture and craniotomy could not be performed. Therefore, it is important to choose a targeted drug treatment.

According to the overall etiological characteristics of intracranial infection in recent years, the distribution is slightly different in different regions and years. The etiology of brain abscesses is still dominated by positive bacteria, and the most common pathogens are *Streptococcus* and *Staphylococcus*. In a retrospective study, 332 patients with brain abscess with positive cultures were counted, and it was found that the most common bacteria were *Streptococcus, Staphylococcus*, and *Proteus* (6).

The positive rate of bacteriological culture in patients with brain abscesses was low. In a 10-year retrospective study, the study found that only 34 cases were positive in pus culture, accounting for 25.76% of the total number of confirmed patients (7). Empirical antimicrobial therapy recommended vancomycin combined with cephalosporins or carbapenems against *Pseudomonas*. However, it should be noted that long-term use of vancomycin and meropenem may cause complications such as neutropenia and imbalance in the bacterial population. The current view is that mNGS has certain advantages over traditional methods in central nervous system infection, improves the positive rate, and shortens the time of diagnosis (8, 9). In this case, *Burkholderia cepacia, S. intermedia*, and *Pseudomonas aeruginosa* were detected in alveolar lavage fluid samples from patient with mNGS, and *S. intermedia* was detected in patient CSF samples. The relative abundance of *Burkholderia cepacia* in bronchoalveolar lavage was higher than in *S. intermedia*. However, *S. intermedia* was detected in the CSF and alveolar lavage fluid, so we determined that *S. intermedia* may be the pathogen, and *Burkholderia cepacia* may be the hospital-acquired infectious bacteria.

Bacteria can invade the brain through direct dissemination or hematogenous dissemination, direct dissemination accounts for 20–60% of intracranial infections, bacteremic dissemination usually causes multiple lesions (1, 10). *S. intermedia* is part of the normal microbiota and are found at various mucosal sites in the respiratory (11). Brain abscesses are frequently caused by oral cavity bacteria (12). The patient had a history of drunkenness and aspiration 1 week before admission. We speculate that the patient's pneumonia due to aspiration, and the *S. intermedia* entered the patient's brain through blood dissemination, resulting in multiple brain abscesses in the patient.

A review of brain abscesses caused by invasive *Streptococcus intermedia* pointed out that most commonly prescribed antibiotic regimens were a combination of ceftriaxone and metronidazole alone (11). Therefore, we stopped vancomycin and meropenem, and switched to ceftriaxone combined with amikacin (Anti-pulmonary infection) for anti-infection treatment.

According to meta-analysis and retrospective study, more central nervous system infections can be correctly treated by mNGS (13–15). Currently, the application of mNGS in central nervous system infections is mainly based on case reports, and there are few large-scale studies to be referred to (16–18).

In summary, we report a patient with multifocal brain abscesses caused by *S. intermedia*; the patient had multiple failed cultures of pathogens from the patient's blood and CSF samples, and the mNGS analysis approach was used, we adjusted the use of antibiotics in time, and finally, the patient achieved good treatment results in a very short time.

## Conclusions

mNGS has certain advantages in identifying brain abscesses; it can effectively avoid delaying the diagnosis and treatment of patients due to the lack of pathogens in routine culture. We believe that mNGS will provide greater help to neurosurgeons in their future work.

## Data availability statement

The original contributions presented in the study are included in the article/supplementary material, further inquiries can be directed to the corresponding author/s.

## Ethics statement

The studies involving human participants were reviewed and approved by the Ethics Committee of the First Affiliated Hospital of Wannan Medical College (Yijishan Hospital). The patients/participants provided their written informed consent to participate in this study. Written informed consent was obtained from the patient's legal representative for the publication of any potentially identifiable images or data included in this article.

## Author contributions

SC and ZY participated in the collection of data and drafted the manuscript. LY collected the data for case presentation. TY reviewed the literature and participated in its design. All authors read and approved the final version of the manuscript.

## Funding

This work was supported by the Key Research and Development Program of Anhui Province (201904a07020034) and the Peak Training Program for Scientific Research of Yijishan Hospital, Wannan Medical College (GF2019G05).

## Conflict of interest

The authors declare that the research was conducted in the absence of any commercial or financial relationships that could be construed as a potential conflict of interest.

## Publisher's note

All claims expressed in this article are solely those of the authors and do not necessarily represent those of their affiliated organizations, or those of the publisher, the editors and the reviewers. Any product that may be evaluated in this article, or claim that may be made by its manufacturer, is not guaranteed or endorsed by the publisher.
